# Outcomes of Kawasaki Disease Children With Spontaneous Defervescence Within 10 Days

**DOI:** 10.3389/fped.2019.00158

**Published:** 2019-04-24

**Authors:** Ya-Chiao Hu, Hsin-Min Liu, Ming-Tai Lin, Chun-An Chen, Shuenn-Nan Chiu, Chun-Wei Lu, Luan-Yin Chang, Jou-Kou Wang, Mei-Hwan Wu

**Affiliations:** Department of Pediatrics, National Taiwan University Hospital and Medical College, National Taiwan University, Taipei, Taiwan

**Keywords:** Kawasaki disease, spontaneous defervescence, coronary artery lesions, immunoglobulin, risk factors

## Abstract

**Background:** Kawasaki disease (KD) is one of the most common vasculitis in childhood. Intravenous γ-immunoglobulin (IVIG) is recommended to be administrated within 10 days after fever onset. However, some patients didn't have IVIG therapies because of atypical disease presentations or spontaneous defervescence. We aimed to evaluate the coronary outcomes of the KD patients who didn't receive IVIG and had defervescence within 10 days.

**Methods:** We retrospectively reviewed the KD patients in NTUCH between 2008 and 2015. The patients with a diagnosis of KD and had a febrile length between 5 and 10 days were enrolled. Days of fever, clinical symptoms, laboratory data at the acute stage, and series of coronary artery measurements within a minimum of 3 months after disease onset were recorded. Risk factors associated with coronary lesions 1 month after KD onset were also analyzed.

**Results:** Two hundred ninety-three eligible KD patients were enrolled (Male: 55.1%, mean age of onset: 1.8 years old). Thirty-seven patients had spontaneous defervescence without IVIG treatment. The incidence of coronary aneurysms at the 4th week after disease onset was higher in spontaneously defervesced KD patients than those treated with IVIG (18.9% vs. 5.1%, *p* = 0.002). Interestingly, of the 238 KD patients without coronary lesions at their acute phase, percentages of emerging coronary aneurysms became significantly higher if they didn't have IVIG therapies due to spontaneous defervescence (4/31), compared with those who received IVIG (3/208). Further analysis showed the development of coronary lesions at 1 month after disease onset was associated with younger age (<12 months old, *p* = 0.024), and leukocytosis (WBC > 17,000/cumm, *p* = 0.031).

**Conclusions:** 18.9% of KD patients with spontaneous defervescence had coronary aneurysms. Even without initial coronary lesions, such patients were still riskier to develop coronary aneurysms, compared with KD patients who received IVIG therapies. Such findings address the importance of refining the strategy for use of IVIG in the spontaneously defervesced KD patients within 10 days after fever onset, at least in those with age younger than 1 year and those with leukocytosis.

## Introduction

Kawasaki disease (KD) is the most common pediatric systemic vasculitis. The incidence in Taiwan is the third highest globally, just lower than that in Japan and Korea (1, 2). The state-of-the-art therapy recommends the intravenous immunoglobulin (IVIG) therapy during the acute stage, which is effective to decrease the coronary complications from 20 to 5% (3). However, within 10 days, some KD patients experienced spontaneous defervescence without IVIG administration. The reasons why they didn't receive IVIG were such as incomplete presentations and consideration of the cost of medication. Although the 2012 guideline of Japanese Society of Pediatric Cardiology and Cardiac Surgery (JSPCCS) recommended clinicians may refrain from IVIG in cases of less severe KD or spontaneous defervescence, based on the considerations detailed in certain scoring systems (4), the risk-stratified management of KD patients who had spontaneous defervescence remains uncertain. Therefore, we aimed to assess the midterm coronary outcomes of Kawasaki disease children with spontaneous defervescence within 10 days and explore the risk factors associated with the development of coronary arterial lesions (CAL) in the subgroup of KD patients.

## Methods

### Study Populations

This study was approved by an ethics committee of National Taiwan University Hospital. In the study, we retrospectively reviewed the institutional databases for patients with a diagnosis of Kawasaki disease at National Taiwan University Children Hospital between January 2008 and December 2015. The diagnosis of KD in this study was made based on clinical criteria for KD (5). Algorithm for the evaluation of incomplete KD patients from the 2004 American Heart Association (AHA) KD guideline (5) was applied to aid the diagnosis. We excluded patients who had defervescence <5 days and over 10 days, who received more than one dose of IVIG, who have <2 principal clinical features, who lost to follow-ups and who had alternative diagnosis eventually, such as adenovirus infection, herpetic gingivostomatitis, and scarlet fever. All patients enrolled in the study received a minimum follow-up period of 3 months in order to evaluate the coronary outcome from the acute phase to the convalescent period. We collected the patients' laboratory data at the acute and subacute phase including hemogram, the serum level of C-reactive protein, sodium, albumin, aspartate transaminase (AST), and alanine transaminase (ALT). Data of urinalysis, urine culture, viral culture, and serum anti-streptolysin O at acute phase were also collected if the patient had received the tests. All of the patients received echocardiography during the acute phase and the subacute phase after fever onset. The frequency of the following echocardiography varied according to the CAL severity. Their laboratory data collected at diagnosis and during follow-ups were reviewed.

### Diagnosis Definition

Based on 2004 guideline of American Heart Association for KD (5), complete KD is diagnosed in the presence of fever for at least 5 days with at least 4 of the 5 principal clinical features. In the presence of <4 principal clinical features, the diagnosis of KD can be made when coronary artery disease is detected by 2D echocardiography. Patients who have fever ≥5 days but do not have sufficient principal clinical findings may be diagnosed with incomplete KD under the support of other clinical, laboratory and echocardiographic findings. In the current study, we defined the patients with a diagnosis of either complete or incomplete KD who had spontaneous alleviation of fever without IVIG treatment within 5–10 days after the onset of disease as “defervesced KD” (dKD).

### Coronary Measurement

The coronary diameters of the left main coronary artery (LMCA), left anterior descending artery (LAD), and right coronary artery (RCA) were collected. The coronary Z score is adjusted by the body surface area and using the reference established from data of Taiwanese children (6). Coronary artery lesion (CAL) is defined as a Z score of coronary diameters ≥ +2.5. (1) On the basis of previous research, persistent CAL for more than 4 weeks after fever onset is defined as coronary artery aneurysm (CA) in this study (7, 8). Coronary aneurysms were sub-classified based on their internal diameter as small (+2.5 ≤ Z < +5.0), medium (+5.0 ≤ Z < 10), and giant (Z ≥ +10.0) (3). CALs, CAs, and the regression were diagnosed based on 2D echocardiography.

### Data Analysis

Patient data were expressed as counts, percentages, medians with interquartile ranges (IQRs) for non-normal distribution data or mean with standard deviation (SD) for normal distribution data. All data analysis was conducted by SPSS statistics (version 22.0) for Windows. We used Chi-square tests and Fisher's exact tests for comparison of categorical variables. The Mann-Whitney U test and Wilcoxon rank-sum test were used for comparison of continuous variables. We set *P* < 0.05 as a statistical significance in this study.

## Results

### Patient Characteristics

A total of 293 cases with KD diagnosis were enrolled in the study. The mean (±SD) age of the enrolled patients was 1.8 ± 1.6 years. Thirty-seven dKD patients (i.e., spontaneous defervescence within 10 days, 12.6%) were identified. The demographics, clinical symptoms, and laboratory features for KD patients treated with IVIG and dKD patients are presented in Table 1. The frequency of incomplete KD in the dKD patients was 51.4%, higher than that in the IVIG-treated group (37%, *p* = 0.11)

**Table 1:** Clinical and laboratory features of KD patients with fever from 5 to 10 days.

	**All (*n* = 293)**	**dKD patients (*n* = 37)**	**Patients with IVIG (*n* = 256)**	***p-*value**
Male gender	161 (54.9%)	20 (54.1%)	141 (55.1%)	0.91
Age **±**SD [range], years	1.8 ± 1.6	2.3 ± 1.7	1.8 ± 1.6	0.06
Total febrile days **±**SD	6.4 ± 1.3	6.3 ± 1.6	6.4 ± 1.3	0.44
Incomplete KD	114 (38.8%)	19 (51.4%)	96 (37.0%)	0.11
Number of principal clinical features,**±**SD [range]	3.7 ± 0.8	3.4 ± 0.8	3.7 ± 0.8	0.04^*^
Disease onset to first echocardiogram, days	6.1 ± 2.6	8.4 ± 4.5	5.7 ± 2.0	0.001^*^
**CLINICAL FEATURES**				
Conjunctival injection	269 (92.4%)	29 (78.4%)	240 (94.5%)	0.001^*^
Skin rash	265 (90.8%)	34 (91.9%)	231 (90.6%)	0.80
Changes in lips and oral cavity	242 (82.9%)	27 (73.0%)	215 (84.3%)	0.10
Extremities change	212 (72.4%)	29 (78.4%)	183 (71.5%)	0.38
Lymphadenopathy	87 (30.3%)	7 (18.9%)	80 (32.0%)	0.11
BCG scar reactivation	108 (53.5%)	14 (37.8%)	94 (57.0%)	0.04^*^
**LABORATORY TEST^a^**				
WBC, ***k*/μ*****L***	14.6 ± 5.1	13.2 ± 4.9	14.7 ± 5.1	0.08
Hb, g/L	11.1 ± 1.1	11.4 ± 0.8	11.0 ± 1.2	0.10
PLT, x**10^4^/μ*L***	350 ± 131	399 ± 144	344 ± 128	0.02^*^
CRP, mg/dL	7.8 ± 6.3	4.5 ± 4.5	8.2 ± 6.3	<0.001^*^
AST, IU/L	79.7 ± 143.5	67.9 ± 106.0	81.2 ± 147.6	0.26
Albumin, g/L	3.8 ± 0.7	4.0 ± 0.4	3.9 ± 0.5	0.10
**CORONARY OUTCOMES**				
CAL at acute phase	54 (18.4%)	6 (16.2%)	48 (18.8%)	0.71
CA at the 4th week	20 (6.8%)	7 (18.9%)	13 (5.1%)	0.002^*^
CAL at any phase	62 (21.1%)	10 (27.0%)	52 (20.3%)	0.35

dKD: KD patients who had spontaneous alleviation of fever without IVIG treatment within 5–10 days after the onset of disease. Values are expressed as percentages (%) and

a *Mean ± SD; SD, standard deviation; BCG, bacille Calmette–Guerin; WBC, White blood cell; CRP, C-reactive protein; AST, Aspartate aminotransferase; Hb, hemoglobin; PLT, platelet; CAL, Coronary artery lesion; CA, coronary artery aneurysm*.

* *Statically significance is defined as p < 0.05*.

Furthermore, of the 37 dKD patients, 18 (48.6%) patients were diagnosed as complete KD. Twelve patients had 4 principle clinical features together with a positive echocardiogram. Two patients had 4 principle clinical features and ≥3 laboratory findings described in the 2004 AHA guideline. Five patients fulfilled only 3 principle features and all of them had a positive echocardiogram. The dKD group presented less number of the five principal clinical features compared to the IVIG group [mean (±SD): 3.4 (±0.8) vs. 3.7 (±0.8), *p* = 0.04]. The dKD group had a significantly lower incidence of conjunctival injection and BCG scar reactivation than the IVIG group. We analyzed the laboratory data collected at the first blood test during the acute phase, platelet count was significantly higher and serum level of C-reactive protein was lower in dKD patients, compared with KD patients with IVIG therapies (Table 1).

### Coronary Outcomes

**Figure 1:**
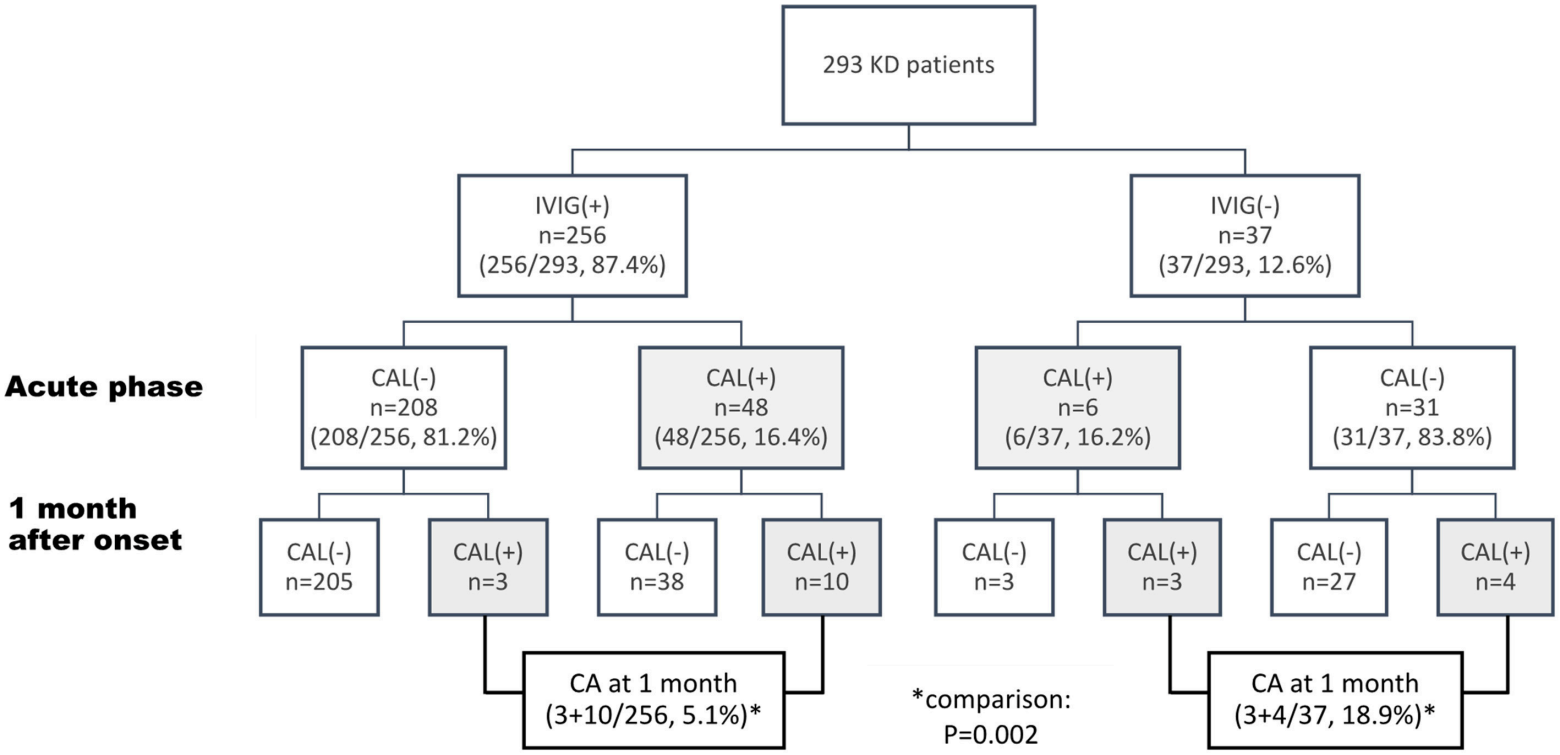
Flow chart of management and coronary outcome of 293 patients with Kawasaki disease (KD) The data are expressed as case numbers (percentage of total patients). IVIG, intravenous immunoglobulin; CAL, coronary artery lesion, defined as Z score of coronary diameters ≥ +2.5.

Of the 293 cases, 54 (18.4%) patients were found to have CAL at their acute phase and 20 (6.8%) developed CA at the 4th week after disease onset. No difference of maximal coronary Z scores or percentages of CAL were noted between IVIG group [mean (±SD):1.65 (±0.99)] and dKD group [1.49 (±0.98), *p* = 0.14] at their acute phase. At 1 month after disease onset, the maximal coronary Z score of dKD group is relatively, not significantly, larger than that of the IVIG group. However, at this time point (1 month), the dKD patients had a higher incidence of CA than the IVIG group (18.9 vs. 5.1%, *p* = 0.002) (Figure 1). Compared with the 256 IVIG-treated KD patients, the 37 dKD patients had their first echocardiography at an average of 8.4 days after fever onset, significantly later than the 256 IVIG-treated KD patients (5.7 days, *p* = 0.001). We did univariate analysis to explore the risk factors of coronary artery lesion at 1 month after KD onset (defined as “coronary aneurysm” in the current study) and found age, incomplete KD, IVIG treatment, changes in lips and oral cavity, white blood cell count, platelet count, and levels of albumin were associated with the development of CA (Supplement Table 1). To avoid interaction of the above risk factors, we conducted multivariate logistic regression and found IVIG treatment (*p* = 0.008) as well as levels of albumin (*p* = 0.002) were the only two independent risk factors for the occurrence of coronary aneurysms.

Of the 37 KD patients, 6 (16.2%), 7 (18.9%), and 2 (5.4%), respectively, had coronary dilatation at 2, 4, and 12 weeks after disease onset (Figure 1, Supplement Figure 1). The most frequently affected coronary arteries was LMCA (*n* = 8, 80%), followed by RCA (*n* = 6, 60%) On the basis of the definition of CA in the current study, 7 (18.9%) patients had CA (small, *n* = 6, median, *n* = 1). Of the 31 dKD patients without initial CAL, four patients (4/31, 12.9%, Figure 1) had coronary progression (to coronary aneurysms) during the first month after KD onset. Of the KD patients without CAL at their acute phase (Figure 1), percentages of emerging coronary aneurysms (at one month after disease onset) became significantly higher if they didn't have IVIG therapies due to spontaneous defervescence (4/31), compared with those who received IVIG (3/208, *p* = 0.006, Figure 1). Coronary aneurysms of the 4 patients regressed at 2.7, 6, 6.8, and 7.3 months after disease onset.

### Risk Factors Associated With Coronary Artery Lesions in Patients With Defervescence Between 5 and 10 Days

Previous studies have demonstrated that coronary severity at 1 month after KD onset was most crucial to the late coronary outcomes. In the current study, 7 patients (18.9%, M:F = 4:3) in the dKD group had coronary lesions at 1 month after fever onset (defined as coronary aneurysms in this study). Table 2 presents the demographic symptoms and laboratory data of dKD patients with (7) and without (30) coronary aneurysms. Univariate analysis showed the development of coronary aneurysms in the dKD group was associated with age (*p* = 0.01) and serum white blood cell count (*p* = 0.03). The percentage of younger than 1 year of age in the 7 dKD patients with coronary aneurysms was 71.4% (5/7), significantly higher than that in the 30 dKD patients without (5/30, *p* = 0.009). The 6 dKD patients with CAL at their acute phase were relatively, not significantly, risky to have coronary aneurysms, compared with 31 dKD patients without (*p* = 0.07). Furthermore, 57% dKD patients with CA (4/7) had WBC count over 17.0 kμ*L*, which was significantly more than the dKD patient without CA (4/30, *p* = 0.027).

**Table 2:** Comparison between spontaneous defervesced KD with and without coronary artery aneurysm 1 month after disease onset.

	**With CA (*n* = 7)**	**Without CA (*n* = 30)**	***P*-value**
Male gender	4 (57.1%)	16 (53.3%)	1.00
Age, years	0.7 (0.5–1.5)	2.2 (1.4–3.8)	0.011^*^
Age < 12 month	5 (71.4%)	5 (16.7%)	0.009^*^
Age < 24 month	7 (100%)	15 (50%)	0.028^*^
Total febrile days	6.5 (5.0–7.0)	6.0 (5.3–7.8)	0.227
Incomplete KD	5 (71.4%)	12 (40.0%)	0.405
Number of principal clinical features	3 (3–3)	4 (2–5)	0.556
CAL at acute phase	3 (42.9%)	3 (10.0%)	0.07
**Laboratory test**			
WBC, ***k*/μ*L***	17.84 (15.32–21.82)	12.10 (9.36–14.89)	0.033^*^
Hb, g/L	10.8 (10.5–10.9)	11.4 (10.9–12.1)	0.173
Hb < 11 g/L	4 (66.7%)	8 (28.6%)	0.154
PLT, x**10^4^/μ*L***	534 (413–616)	408 (276–479)	0.091
CRP, mg/dL	5.04 (4.14–5.23)	1.98 (1.15–6.74)	0.408

*Values are expressed as medians (IQRs) or number and percentages (%); WBC, White blood cell; Seg, Neutrophil segment; CRP, C-reactive protein; AST, Aspartate aminotransferase; Hb, hemoglobin; PLT, platelet*.

* *Statically significance is defined as p < 0.05*.

To avoid interaction between age and WBC count, we conducted multivariate logistic regression to identify the independent risk factors of developing coronary aneurysms in the 37 dKD patients. The results showed that age [younger than 1 year, odds ratio: 24.6, 95% confidence interval (CI) = 1.5–399, *p* = 0.024] and serum white blood cell count more than 17.0 kμ*L* (odds ratio: 16.1, 95% CI = 1.3–198, *p* = 0.031), rather than CAL at the acute phase, were both independently associated with the occurrence of coronary aneurysms in the dKD patients.

## Discussion

IVIG therapy remained the standard treatment for KD, which can effectively reduce the risk of CAL development. However, in the cases with spontaneous defervescence, treatment guidelines were still uncertain because of short of well-established data of their clinical outcomes. In this retrospective cohort, we found that 12.6% of KD patients had spontaneous defervescence within 10 days and 18.9% of such patients (dKD) suffered from coronary aneurysms, significantly higher than the percentages of coronary aneurysms in the IVIG group (5.1%, *p* = 0.002, Table 1, Figure 1). Such findings reinforce the importance of IVIG in the treatment of KD. We also demonstrated, in the KD patients without CAL at their acute phase, percentages of emerging coronary aneurysms (at 1 month after disease onset) became significantly higher if they didn't have IVIG therapies due to spontaneous defervescence (4/31), compared with those who received IVIG (3/208). Such observation had never been described before and drove us to look for the potential risk factors of developing coronary aneurysms in the dKD patients.

We demonstrated that, among the 37 dKD patients, younger age (<1 year old) and leukocytosis were associated with the development of coronary aneurysms. Downie et al. reported that males, age <1-year-old and higher platelet count were associated with increased odds of CA formation for patients with delayed or no treatment compared to KD patients with prompt IVIG (9). Younger KD patients were thought of being less likely to have a complete presentation of KD and also suffered from an increased risk of CAL (10–12). For example, Salgado et al. showed percentages of CAL and incomplete KD were significantly higher in the KD patients younger than 6 months old, compared with those were older. Moreover, 18.6% of patients <6 months old with normal echocardiogram initially developed CAL within 8 weeks of diagnosis (13). Our data presented similar findings and younger age remained an important risk factor of CAL even though the patient had spontaneous defervescence. Taken the above together, for the KD patients younger than 1 year old, we may recommend the use of IVIG even if they have defervescence without IVIG therapy because of increased risk of CAL.

Spontaneous defervescence without IVIG is not new for the KD patients. Depending on the definition of spontaneous defervescence, the percentages ranged from 7.3 to 20.9% (7, 14, 15). Takahashi et al enrolled patients who had spontaneously defervescence within 7 days, rather than 10 days as our cohort, and their results showed 7.3% of 968 KD patients had fever persistent <7 days without an infusion of IVIG. If we applied the same enrollment criteria as Takahashi et al. (14), to our current cohort, the percentage of spontaneous defervescence would decrease from 12.6 to 10.5%. Spontaneous defervescence in KD patients usually causes hesitation of physicians and parents on the use of IVIG. However, Takahashi et al. showed 11.2% of KD patients with spontaneous defervescence within 7 days after disease onset may have recurrent fever 3–7 days later and predispose to develop coronary artery lesions (14). The current study further innovatively points out, even without initial CAL and recurrence of fever, such subgroup of KD patients still carried a significantly higher risk of late development of coronary aneurysms, compared with those had IVIG therapies (4/31 vs. 3/208, *p* = 0.006). Both age (younger than 1 year) and leukocytosis were independently associated with the development of coronary aneurysms in the dKD patients. In summary, it's time to refine the strategy for use of IVIG in the spontaneously defervesced KD patients within 10 days after fever onset, at least in those with age younger than 1 year and those with leukocytosis.

Studies in the 1990s (16, 17) demonstrated, in the KD patients without IVIG treatment, levels of TNF alpha and IL-2 continued increasing even at the second or third week after fever onset. Until 2–3 months later, levels of the two cytokines decreased gradually to the normal ranges. TNF alpha and IL-2 were two of the key inflammatory cytokines in the pathogenesis of KD and associated with recruitment of immune cell population, T cell activation, and CAL development (18). Together the above, for the KD patients with spontaneous resolution of fever within 10 days, delayed normalization or even increase in levels of cytokines, like TNF alpha and IL-2, may accompany higher risk to the development of coronary lesions.

There were several limitations in this study. First, there was selection bias especially when we enrolled the KD patients with atypical disease presentations. To minimize the bias, we used the evaluation algorithm for suspected incomplete KD according to AHA guideline (3) and traced their clinical presentation as well as laboratory data in the subacute phase to aid diagnosis. Second, the number of dKD patients was small which made the statistical analysis limited. Third, information bias might exist, since the ultrasound technicians were not blind to tentative or previous diagnosis. Finally, because of the observational nature of our study, large-scaled prospective studies are necessary to determine the efficacy and necessity of IVIG in dKD patients who had higher risk to develop coronary abnormalities.

## Conclusions

18.9% of KD patients with spontaneous defervescence had coronary aneurysms. Even without initial coronary lesions, such patients were still riskier to develop coronary aneurysms, compared with KD patients who received IVIG therapies. Such findings address the importance of refining the strategy for use of IVIG in the spontaneously defervesced KD patients within 10 days after fever onset, at least in those with age younger than 1 year and those with leukocytosis.

## Author Contributions

M-TL and M-HW contributed to the conception and design of the study. H-ML and L-YC organized the database. Y-CH and M-TL performed the statistical analysis. Y-CH wrote the first draft of the manuscript. C-AC, S-NC, C-WL, and J-KW took care of patients and collected data. M-TL had full data access and is accountable for all aspects of the work in ensuring that questions related to the accuracy or integrity of any part of the work are appropriately investigated and resolved. All authors contributed to manuscript revision, read and approved the submitted version.

### Conflict of Interest Statement

The authors declare that the research was conducted in the absence of any commercial or financial relationships that could be construed as a potential conflict of interest.
