# Supplementary material for: Outcomes of Kawasaki Disease Children With Spontaneous Defervescence Within 10 Days
Source: Front Pediatr. 2019 Apr 24;7:158. doi: 10.3389/fped.2019.00158 (PMC6491630; doi:10.3389/fped.2019.00158)
Supplement: Supplementary file 1 [file Table_1.DOCX]

Supplementary Materials

| Supplement Table 1.  Clinical and laboratory features of KD patients with and without coronary aneurysms (CA) | | | | | | | |
| --- | --- | --- | --- | --- | --- | --- | --- |
|  | All  (n=293) | | Patients without CA  (n=273) | | Patients with CA (n=20) | | *p* value |
| Male gender | 161 | (54.9%) | 151 | (55.3%) | 10 | (50%) | 0.645 |
| Age$\boldsymbol{\pm}$SD [range], years | 1.8$\pm$1.6 | | 1.9$\pm$1.6 | | 1.2$\pm$1.2 | | 0.024* |
| Total febrile days$\boldsymbol{\pm}$SD | 6.4$\pm$1.3 | | 6.3$\pm$1.3 | | 7.0$\pm$1.7 | | 0.066 |
| Incomplete KD | 114 | (38.8%) | 101 | (37.0%) | 14 | (70.0%) | 0.004* |
| Treated with IVIG | 256 | (87.4%) | 243 | (89.0%) | 13 | (65.0%) | 0.002* |
| Number of principal clinical features,$\boldsymbol{\pm}$SD [range] | 3.7$\pm$0.8 | | 3.7$\pm$0.8 | | 3.4$\pm$0.8 | | 0.060 |
| Disease onset to first echocardiogram, days | 6.1$\pm$2.6 | | 6.0$\pm$2.6 | | 6.9$\pm$2.5 | | 0.050 |
| Clinical features |  |  |  |  |  |  |  |
| Conjunctival injection | 269 | (91.8%) | 250 | (91.6%) | 19 | (95.0%) | 0.590 |
| Skin rash | 265 | (90.4%) | 248 | (90.8%) | 17 | (85.0%) | 0.391 |
| Changes in lips and oral cavity | 242 | (82.9%) | 231 | (84.9%) | 11 | (55.0%) | 0.001* |
| Extremities change | 212 | (72.4%) | 196 | (71.8%) | 16 | (80.0%) | 0.428 |
| Lymphadenopathy | 87 | (29.7%) | 82 | (30.0%) | 5 | (25.0%) | 0.634 |
| BCG scar reactivation | 108 | (36.9%) | 97 | (35.5%) | 11 | (55.0%) | 0.081 |
| Laboratory test^a^ |  |  |  |  |  |  |  |
| WBC, $\mathbf{k/}\boldsymbol{\mu L}$ | 14.6$\pm$5.1 | | 14.4$\pm$5.0 | | 17.5$\pm$5.3 | | 0.012* |
| Hb, g/L | 11.1$\pm$1.1 | | 11.1$\pm$1.1 | | 10.7$\pm$1.2 | | 0.269 |
| PLT, x$\mathbf{10}^{\mathbf{4}}\mathbf{/}\boldsymbol{\mu L}$ | 350$\pm$131 | | 342.6$\pm$123.4 | | 467.8$\pm$174.0 | | 0.001* |
| CRP, mg/dL | 7.8$\pm$6.3 | | 7.7$\pm$6.3 | | 8.7$\pm$5.9 | | 0.404 |
| AST, IU/L | 79.7$\pm$143.5 | | 81.6$\pm$148.0 | | 55.8$\pm$55.3 | | 0.855 |
| Albumin, g/L | 3.8$\pm$0.7 | | 3.9$\pm$0.5 | | 3.6$\pm$0.5 | | 0.009* |

Values are expressed as percentages (%) and aMean$\pm$SD ; SD: standard deviation, BCG: bacille Calmette–Guerin; WBC: White blood cell, , CRP: C-reactive protein, AST: Aspartate aminotransferase, Hb: hemoglobin, PLT: platelet
* Statically significance is defined as p< 0.05

**Supplementary Table 2.** Clinical characteristics and coronary outcome in dKD patients with Coronary aneurysm

| Case | | Age, years | Sex | Febrile days | KD criteria | Reason for not using IVIG | Max. z-score in acute phase (DOI) | Max. z-score at 1month | Outcome |
| --- | --- | --- | --- | --- | --- | --- | --- | --- | --- |
| 1 | 0.62 | | M | 5 | 4/5 | fever subsided, no CAL initially | 2.55(9)^#^ | 2.89 | CRP: Reg. in 5.2 months |
| 2 | 1.90 | | F | 8 | 3/5 | fever subsided, no CAL initially | 2.36(11) | 2.58 | Reg. in 2.7 months |
| 3 | 0.70 | | M | 5 | 3/5 | fever subsided, no CAL initially | 2.04(4)^*^ | 3.52 | Reg. in 6 months |
| 4 | 0.40 | | M | 10 | 3/5 | 2 criteria met at acute phase | 2.49(6)^⧫^ | 3.8 | Reg. in 6.8 months |
| 5 | 0.34 | | M | 5+3 | 4/5 | fever subsided, no CAL initially | 0.91(6) ^◎^ | 4.8 | Reg. in 7.3 months |
| 6 | 1.90 | | F | 7 | 3/5 | 2 criteria met at acute phase | 2.75(5) | 4.31 | Reg. in 2 months |
| 7 | 0.96 | | F | 6 | 3/5 | 2 criteria met at acute phase | 2.67(10) | 5.07 | Reg. in 7 months |

*DOI* days of illness, *Reg* regression
# no CAL before 5^th^ DOI at other hospital, * no CAL at 1^st^ measurement and CAL noted at 19^th^ DDOI

⧫ CAL (Max. z-score:4.49) noted at 22^th^ DOI,
◎ no CAL at 1^st^ measurement and CAL (Max. z-score: 3.48) noted at 15^th^ DOI

FIGUR LEGEND

**Supplementary Figure 1** Serial maximal coronary Z score in the 7 dKD patients with coronary aneurysm at 1 month after disease onset
