# Supplementary figures and images for: Outcomes of Kawasaki Disease Children With Spontaneous Defervescence Within 10 Days
Source: Front Pediatr. 2019 Apr 24;7:158. doi: 10.3389/fped.2019.00158 (PMC6491630; doi:10.3389/fped.2019.00158)

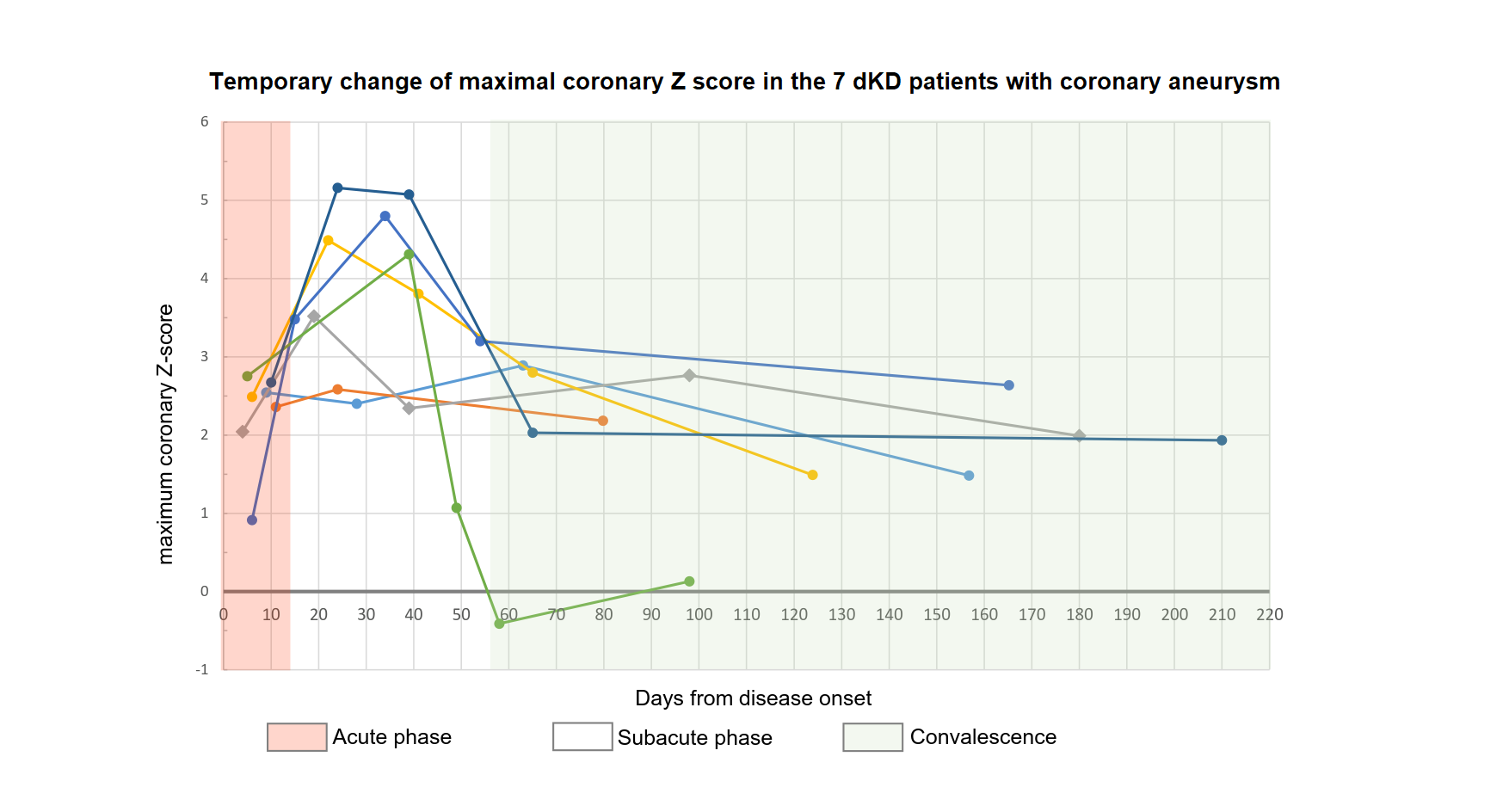

Supplement: Supplementary file 2 [file Image_1.tif]
